# A framework for optimising arthropod DNA quality and quantity for modern sequencing tools using hard ticks (Ixodidae)

**DOI:** 10.1007/s00436-025-08583-0

**Published:** 2026-01-06

**Authors:** X. W. Barton, S. S. Tobe, J. B. Fontaine, C. L. Oskam

**Affiliations:** 1 School of Medical, Molecular and Forensic Sciences, College of Environmental and Life Sciences, Murdoch University, Perth, 6150 Australia; 2 Centre for One Health and Biosecurity, Harry Butler Institute, Murdoch University, Perth, 6150 Australia; 3 School of Environmental and Conservation Sciences, College of Environmental and Life Sciences, Murdoch University, Murdoch University, Perth, 6150 Australia

**Keywords:** High molecular weight DNA, Tissue disruption, DNA fragmentation, DNA yield, Hard ticks (Ixodidae)

## Abstract

Advanced sequencing technologies require strict standards for DNA input and integrity. This study addresses the challenge of extracting high-quality, endogenous DNA from smaller arthropods with mixed DNA sources (arthropod, host, and microbiome), using *Amblyomma triguttatum* as a model organism. We evaluated three tissue types (Whole, Bisected, and Legs), three disruption methods (Undisrupted, Sliced, and liquid nitrogen bead Homogenisation), and two extraction kits (Qiagen DNeasy Blood & Tissue and MagAttract HMW) to optimise DNA yield, quality, and composition. The Qiagen MagAttract High Molecular Weight Kit significantly increased the proportion of large DNA fragments (20–48.5 kbp) by 11-fold compared to the Qiagen DNeasy Blood & Tissue Kit. Aggressive homogenisation methods produced the highest proportion of short fragments (97%, 1–10 kbp). Whole-Homogenised specimens yielded the highest DNA concentration (198 ng µL⁻¹), whereas Bisected-Undisrupted specimens achieved 146 ng µL⁻¹ with a greater proportion of large fragments (3.15%). Bacterial DNA content remained consistent across treatments. Our findings highlight the importance of selecting appropriate extraction methods to ensure optimal DNA quality for advanced sequencing applications. These results provide useful guidelines for optimising DNA extractions from smaller-bodied arthropods (~ 10–20 mg) and establish a framework for future studies to consider DNA quantity, quality, and composition.

**Graphical Abstract:**

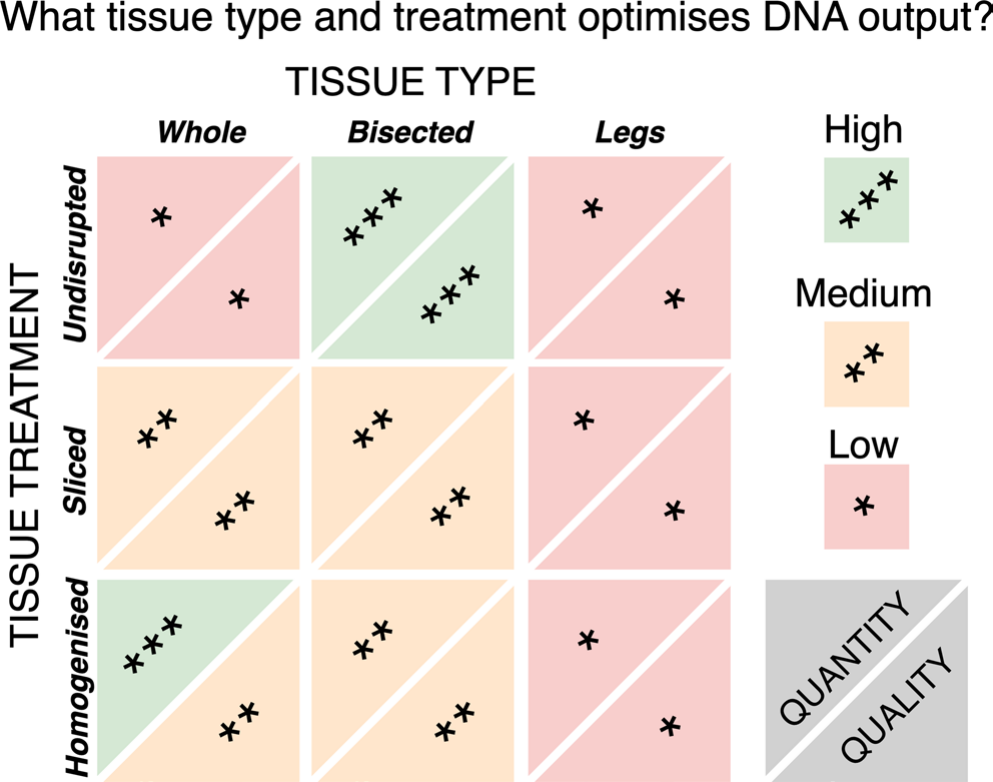

## Introduction

Vectors of disease pose major challenges and concerns for the health and well-being of people, livestock and companion animals (Jongejan and Uilenberg 2004). Globally, billions of dollars are spent annually on preventing and treating vector-borne diseases (Hurtado et al. 2018; Mac et al. 2019). These risks and exposures are expected to increase as climate changes and human land use continues to expand (Gray et al. 2009; Nuttall 2021). A major class of disease vectors are haematophagous arthropods, which transmit pathogens that cause diseases such as Lyme (ticks), malaria, dengue fever (mosquitoes) and Chagas (triatomine bugs) (de Souza and Weaver 2024). Quantification and disease risk forecasting increasingly depend on molecular techniques that seek to characterise both pathogen and host dynamics.

The past 30 years have seen a rapid shift from simple PCR to advanced sequencing methods, accompanied by major changes in DNA input requirements. Advances across the three generations of sequencing technologies, including Sanger, short-read, and long-read methods, have greatly expanded the capacity to characterise pathogens and organisms with complex life histories and to process larger numbers of samples more efficiently (Kneubehl et al. 2022; Egan et al. 2021). However, these tools all rely upon inputs of appropriate DNA quality and quantity.

Integrity of downstream sequence data is dependent on the quantity and quality of DNA extractions contingent on the sequencing platform. For instance, PCR only requires a small amount of DNA to obtain results, enabling the amplification of fragmented and low concentration DNA. Comparatively, short-read second generation sequencing (formerly next generation sequencing, NGS) requires specific inputs of between 1 and 500 ng of DNA (Illumina 2016) and are optimised for reads less than 500 bp, allowing for highly fragmented DNA (Tan et al. 2019). Long-read sequencing (third generation) requires significantly longer and higher DNA inputs to obtain informative results, creating a need for application specific DNA extraction protocols (Barton et al. 2025). As DNA extractions are required to be optimised for specific sequencing platforms, failing to do so can result in wasted time, money, and resources. Although these standards are needed, there is still a lack of protocols that enable optimal DNA purification from arthropods.

Ixodidae (hard ticks) possess a four-instar life cycle (eggs, larvae, nymphs, and adults), typically feeding on up to three different vertebrate hosts. Thus, their dynamics and ecology are intertwined with their hosts with direct and indirect ramifications of factors such as livestock trade, wildlife movements, urbanisation, and changing climactic conditions (Oorebeek and Kleindorfer 2008; Backus et al. 2021).

Such complexity presents major challenges in monitoring and understanding pathogen transmission risk, underscoring the need for innovative genomic surveillance tools. A broad range of genomic research has been applied to ticks and the pathogens they transmit, utilising various sequencing technologies. Traditional methods like Sanger sequencing have allowed highly specific and targeted identifications of tick species and their pathogens (Greay et al. 2018; Páez-Triana et al. 2021; Myers and Scimeca 2024). Higher-throughput platforms, such as short-read sequencing and long-read sequencing platforms like Oxford Nanopore, allow for the detection of a more diverse array of pathogens, including potential novel or previously unseen pathogens in a sampling region (Lado et al. 2019; Guerrero et al. 2019; Egan et al. 2021; Kneubehl et al. 2022).

Despite extensive research on the biology of ticks, there remains a significant gap in optimising DNA purification methods from these arthropods. While numerous studies have explored DNA extraction methods on ticks, most have relied primarily on PCR-based experiments to evaluate their effectiveness (Halos et al. 2004; Ammazzalorso et al. 2015; Miura et al. 2017). Much of the published literature discussing the DNA extraction of ticks is prior to the conception of advanced sequencing technologies and may no longer apply to the wide range of currently available extraction kits and sequencing methods (Hill and Gutierrez 2003; Halos et al. 2004). Additionally, while most gDNA in unfed ticks comes from the tick itself, DNA from the host or microbiome can complicate the interpretation of tick genomic data in studies targeting tick DNA.

Given rapidly evolving tools, there is an urgent need for up-to-date standardised methods to optimise DNA quality, quantity, and composition for sequencing small (~ 10–20 mg) arthropods such as ticks. Therefore, our aims are: (1) to determine the spatial distribution of DNA within tick specimens using microscopy; (2) to assess the impact of tissue disruption and type on DNA yield; (3) to evaluate how these factors influence DNA fragmentation; and (4) to determine their effect on bacterial load in DNA purifications. These assessments provide researchers with standardised, optimised DNA extraction protocols for arthropods with a biomass comparable to ticks (~ 10–20 mg), tailored for advanced sequencing technologies and adaptable to arthropods of broadly similar sizes. This approach minimises the time, financial, and material resources typically required for troubleshooting extraction methods for challenging arthropod samples.

## Materials and methods

### Sample collection

An ideal model organism for testing modern tools and their optimisation will be relatively small bodied, potentially difficult to process, and have non-target DNA contamination potentially related to questions of interest (i.e., disease). We selected *Amblyomma triguttatum* (ornate kangaroo tick) as our study organism due to its small body size compared to larger arthropods with higher biomass (e.g., many beetles, stick insects, spiders, and scorpions). This species also features a hard chitinous exoskeleton, is known to host a diverse microbiome, and harbours disease-causing pathogens (*Coxiella burnetii*) (Graves and Stenos 2017; Egan et al. 2021) and contains a mixed source of DNA (endogenous, host and microbial). To maintain uniformity between samples, a single instar was selected, with adult female *Amblyomma triguttatum* used for all specimens. This was done to standardise biomass across samples and to minimise the potential confounding effects of microbiome differences between instars (Egan 2022).

Specimens were collected using the dragging method from various sites within the Swan Coastal Plain surrounding the capitol, Perth (−31.02°−33.42°S, 115.64° 116.05°E), in Western Australia (Salomon et al. 2020). Dragging involves moving a 1 × 1 m white flannel cloth through the environment to which ticks will attach. Ticks were also collected opportunistically from specimens found on clothing in the field. Ticks were preserved in 70% ethanol and stored at 4 °C to balance morphological and DNA integrity (Marquina et al. 2021), specimens used in this study were stored between 6 months and 1 year in these conditions. Ticks were identified morphologically to life stage and species using taxonomic keys and species descriptions (Roberts 1970; Barker and Walker 2014).

### Microscopy

To characterise DNA distribution within specimens, we used microscopy with staining. Four adult female *A. triguttatum* ticks were processed based on the protocol outlined by Lee (2019). Specimens were dehydrated with ethanol, embedded in paraffin wax, sectioned using a microtome (4 μm), and placed onto positively charged glass microscope slides (Hurst Scientific). Subsequently, sections were subjected to staining using 1X Diamond™ Nucleic Acid Dye (Promega) and 1X hematoxylin & eosin (H&E) to visualise the spatial distribution of DNA using a Nikon Optiphot with fluorescent attachment using a B2 filter.

## Experimental design and sample preparation

### Tissue type and preparation

Given the multiple potential DNA sources within ticks (tick, host, and microbiome), selecting specific tissue types may be beneficial depending on the desired application. Adult female *A. triguttatum* specimens were categorised into three distinct tissue type groups for analysis. The first group comprised whole ticks (Whole), which were left intact. This approach potentially increases DNA yield while preserving the gut microbiome. The second group consisted of bisected ticks (Bisected), where specimens were halved, mid-sagittal, using a sterile scalpel (shape #11). DNA was extracted from one half of the specimen, leaving the other half intact for future morphological or molecular analyses. The final group included tick legs (Legs), isolated by detaching the legs from the body using sterile forceps. It was anticipated that using only legs may reduce microbial load, attributed to the absence of gut material in the extracted tissue.

#### Mechanical tissue treatment

**Fig. 1:**
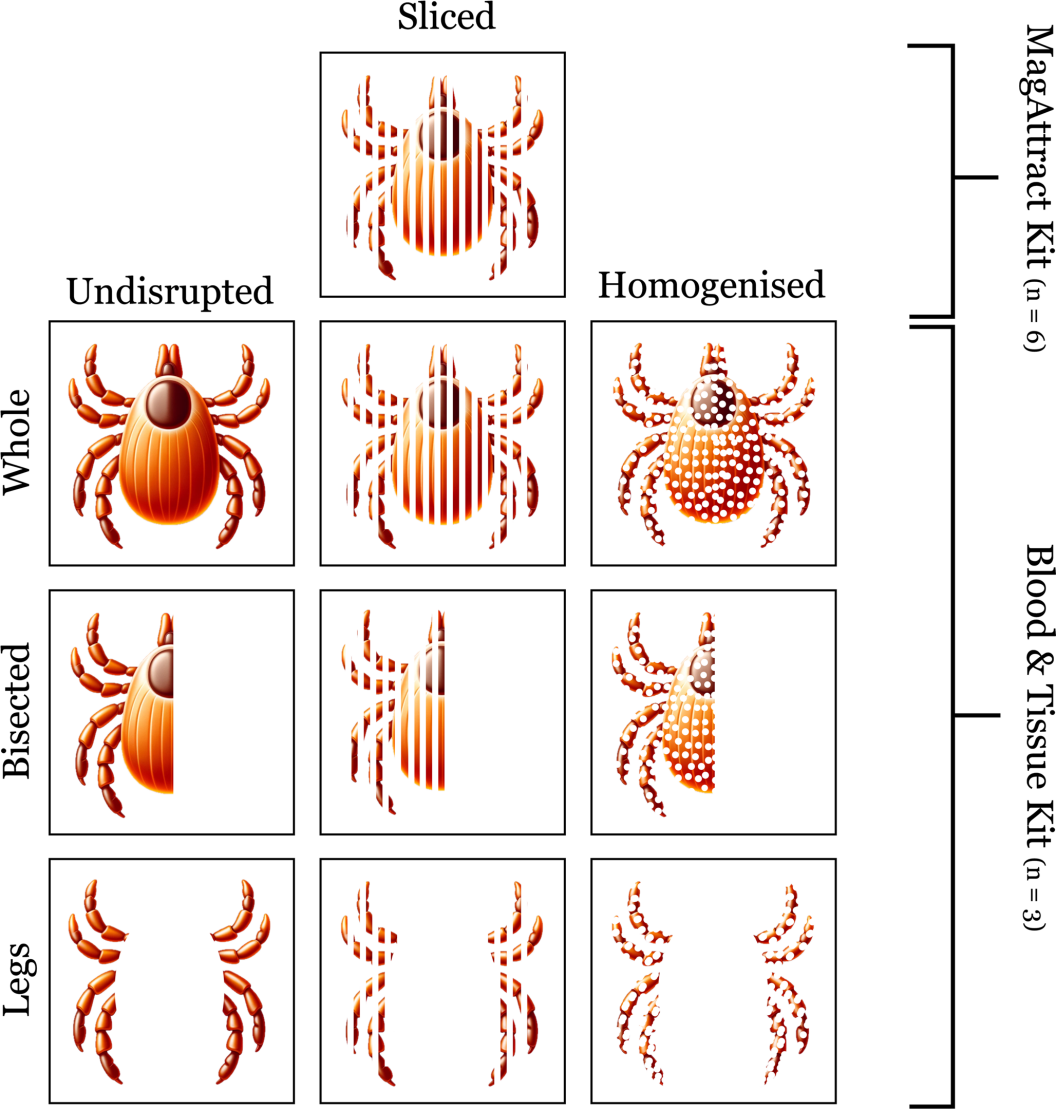
Experimental design (*n* = 3 *Amblyomma triguttatum* specimens per treatment combination) where tissue type and tissue disruption were varied prior to DNA extraction using Qiagen DNeasy Blood & Tissue Kit and Whole-Sliced samples were tested using Qiagen MagAttract HMW Kit (*n* = 6)

Tissue preparation methods impact DNA yield and fragmentation, affecting the suitability of DNA purifications for sequencing applications that require different input thresholds. Thus, to assess trade-offs, tissue samples were subjected to tissue disruption using one of three methods. These ranged from techniques aimed at minimising DNA fragmentation to more disruptive approaches designed to maximise DNA yield. First, the tick tissues were left whole for proteolytic digestion (Undisrupted); second, tick tissues were sectioned into roughly 0.5–1 mm pieces using a shape 11 scalpel blade (Sliced); and third, tick tissues were homogenised using ‘bead beating’ by freezing with liquid nitrogen in a 2 mL SafeLock Eppendorf tube for 5 min and homogenised with a single 3 mm stainless steel bead (Qiagen) on a Qiagen TissueLyser LT at 50 oscillations per second for 1 min (Homogenised). For each unique tissue treatment and extraction kit combination, we included three adult female *A. triguttatum* individuals (*N* = 27 in total) and one extraction blank per treatment (Fig. 1).

**Fig. 2:**
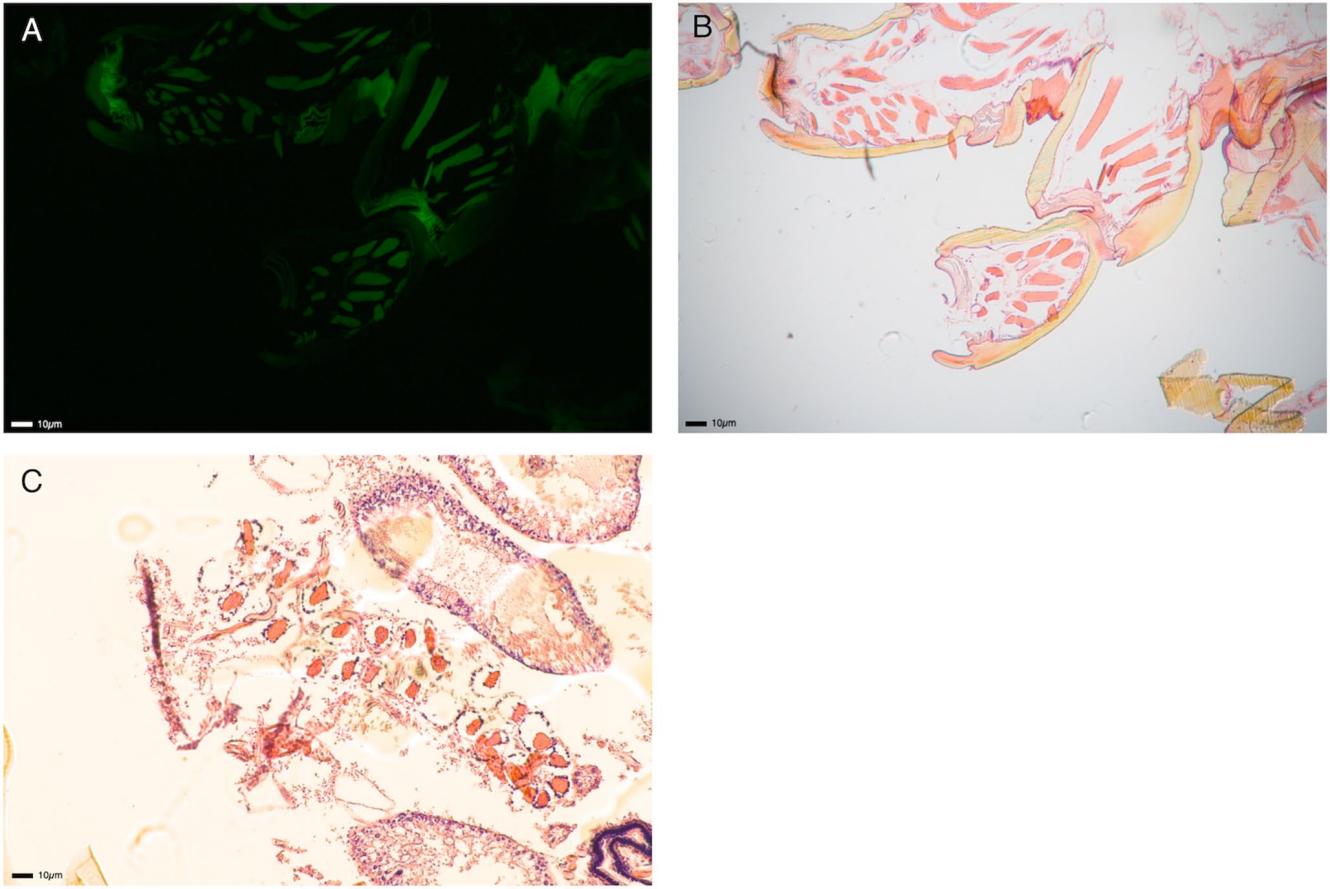
**A**: Autofluorescence of *A. triguttatum* leg/coxae under UV light with H&E stain, **B**: Section of *A. triguttatum* leg/coxae with H&E staining under visible light, section A and B being the same section. **C**: Section of *A. triguttatum* gut region with H&E staining under visible light. Dark purple staining indicates DNA/nucleus

### DNA purification

#### Qiagen DNeasy blood & tissue kit

DNA extraction was performed using a Qiagen DNeasy Blood & Tissue Kit (Qiagen, Germany), using a modified version of the Hair and Nails protocol (Qiagen 2006) with the following changes. After mechanical tissue preparation, samples were incubated in a lysis buffer made up of 180 µL of buffer ATL and 20 µL of proteinase K at 56 °C for ~ 16 h. For DNA elution from spin column, Buffer AE (25 µL) was added to the spin column, incubated for 1 min at room temperature, and then centrifuged at 6000 X g for 1 min. This step was repeated once more with another 25 µL of Buffer AE, resulting in a final elution volume of 50 µL.

#### Qiagen magattract high molecular weight (HMW) kit

After analysing the results from the Qiagen DNeasy Blood & Tissue kit, the most suitable tissue preparation and type were established. To evaluate potential further improvements, six additional individuals were processed using a Qiagen MagAttract DNA (Qiagen, Germany) purification kit. Specimens were Whole-Sliced using the methods described above (Figure. 1). The same lysis conditions were used as per Qiagen DNeasy Blood & Tissue Kit.

protocol. Samples were then incubated with 4 µL of RNase A and incubated at room temperature for 2 min. Next, 150µL of Buffer AL and 280 µL of Buffer MB were added and incubated on a thermoblock at room temperature for 3 min at 1400 rpm. Tubes were placed on a 3D printed MagSep rack (Thingiverse user ‘cmmonaco’ https://www.thingiverse.com/thing:4939805) for 2 min. The supernatant was then removed, and tubes were then removed from the magnetic rack. Buffer MW1 (700 µL) was added, and tubes were incubated for 2 min a 1400 rpm. Tubes were then placed back onto the magnetic rack for the beads to separate. A second wash of MW1 and two washes with Buffer PE were performed in the same manner. Tubes were then left of the magnetic rack and 700 µL of water was added, removed, and then repeated. Tubes were then removed from the magnetic rack and beads were eluted in 100 µL of Buffer AE. Samples were incubated for 3 min at 1400 rpm and placed back on the magnetic rack. Eluted gDNA was then transferred to 1.5 mL Lo-Bind SafeLock Eppendorf tubes.

### DNA quantification and quality assessment

The performance of each DNA extraction method was assessed in three ways: firstly, DNA quantity was tested using a Qubit 2.0 Fluorometer; secondly, using an Aligent 2200 TapeStation to determine level of DNA fragmentation; and finally, qPCR was performed to determine relative quantities bacterial DNA present in the gDNA extracts, calculated to number of copies using gBlocks (Conte et al. 2018).

#### Qubit

First, to detect low DNA concentrations, samples were assessed using Qubit High Sensitivity (HS) assays. If samples contained concentrations of DNA that were too high for Qubit HS assays, these samples were retested using the Qubit Broad Range (BR) assay. The Qubit assays were performed by pipetting 198 µL of HS or BR working solution and 2 µL of gDNA extract into thin wall 500 µL Ultraclear Qubit Assay Tubes. Two Qubit standards were made up from 10 µL of Standard 1 and Standard 2 and 190 µL of HS or BR working solution. Samples were incubated at room temperature for 5 min and measured on a Qubit 2.0 Fluorometer.

#### TapeStation

For each sample, 2 µL of gDNA was sent to the Australian Genome Research Facility (AGRF; Perth, Western Australia) and DNA fragmentation was assessed on an Aligent DNA TapeStation. XML and CSV files were then exported from the Aligent TapeStation 5.1 Software for Windows 10 and imported into RStudio using the package BioanalyzeR (https://github.com/jwfoley/bioanalyzeR).

#### qPCR

The qPCR assays were prepared using the KAPA SYBR FAST master mix (Roche, Switzerland), following the manufacturer’s instructions. Each reaction totalled 25 µL containing 2x KAPA SYBR FAST qPCR master mix, 0.425 mM of forward and reverse primer (Table 1), ultrapure DNA-free water, and 2 µL of template DNA. The qPCR assays were performed on a Qiagen RotorGeneQ using the Qiagen RotorGeneQ software for Windows 10 under the following cycling conditions: initial denaturation at 95 °C for 5 min, followed by 40 cycles of denaturation at 95 °C for 30 s, annealing 55 °C for 30 s and extension at 72 °C for 30 s, and a final extension of 72 °C for 5 min. A standard curve for quantification was generated using a serial dilution of a gBlock (Integrated DNA Technologies, Inc., USA, IDT) (Table 2) specifically designed for bacterial primer sets. gBlocks were prepared according to IDT recommendations by resuspending with AE buffer in Qiagen Lo-Bind Eppendorf tubes.

**Table 1:** Primers used for 16 S bacterial qPCR

Primer Name	Size (bp)	Target taxa	Target Gene	Sequence (5’ – 3’)	Reference
*27 F-Y*	250–320	Bacteria	16 S rRNA V1-2	AGAGTTTGATCCTGGCTCAG	(Gofton et al. 2015)
*338R*	250–320	Bacteria	16 S rRNA V1-2	TGCTGCCTCCCGTAGGAGT	(Turner et al. 1999)

**Table 2:** gBlock sequence used for qPCR standardisation (primer binding sites in bold) gBlock

gBlock Sequence (5’ – 3’)
tgcatgatctacgtgcgtcacatgcagtac**AGAGTTTGATCCTGGCTCAG**CGAATTCCCAGGTTAAGCTGTCACTCCACACCGTTCCGCTAACGGAGTGTACTCATGGCTGGCCCCCTCTGAAGATTTCCACAAGCAGTCAGGCGTGAGCTATATTCTGAAGGATCACCGCTAGTGGACCAGTCTTGGACGCGACCGCCCGGACTTGAGAGAGTACCCCTATCTAAGATTGTCTGAGTTGTCCGTTGCTTGTGTGCCAATGTAAAGCATCTATTTTCAAAAGGTGCGGTGCCCAATCGGAGCGCGGCTCCGGATGGCGTCCCCCCCTGCCTAGGGTGGGAGGGGTAATAT**ACTCCTACGGGAGGCAGCA**ctgaccagagatttcctacg

gBlocks were received lyophilised and resuspended to 10 million copies per µL based on the molecular weight of the fragment and the yield. These were further diluted to concentrations from 10 million copies to 0.01 copies. The standard curve was generated using four replicates of 0.01, 0.1 and 10 million copies and eight replicates of 1–1 million copies. Triplicates of the replicates of each gBlock copy number were subset and combined to create 10 million unique standard curves using CurveLooper (https://github.com/XWBarton/CurveLooper). A minimum, maximum, median, and mean R, R2 and regression line were calculated from these standard curves to assess the possible variations in gBlock copy number fit. Once the standard curves were generated, each sample, two no template controls, and two of each dilution of gBlock underwent the same qPCR conditions.

### Data analysis

To evaluate the quantity, quality, and composition of the gDNA extractions for each treatment, we visualised our data and applied linear regression to evaluate strength of evidence. We used 0.05 as our threshold for inferring evidence of a statistical difference among treatments. For each analysis we report the effect estimate, standard error, t or z value, and p-value. To perform analyses, we used R and RStudio and the package lme4 for mixed effect modelling (Bates et al. 2014), lmerTest for obtaining p values (Kuznetsova et al. 2017) and ggplot2 for data visualisation (Wickham 2011).

For DNA quantity, quality, and composition experiments we had a shared model structure with our response variable (quality, quantity, bacterial content) and model predictors tissue disruption method (Undisrupted, Sliced, or Homogenised), tissue type (Whole, Bisected or Legs) and their two-way interactions. Our reference treatment was Whole-Homogenised due to it being the most common sample preparation method for tick gDNA extractions. Our dataset was made up of *n* = 27 unique ticks. To address the small sample size, we employed mixed effects modelling, which allowed us to account for variation between individual ticks and tissue weight, thereby providing more reliable estimates of DNA yield while presenting both absolute and normalised values for comparison across treatments.

For quantity, each unique extraction was measured in triplicate. We used all observations and applied a mixed effect model with fixed effects of treatment and tissue quantity (mg) and random effect of individual tick given triplicate observations. We followed modelling suggestions as outlined in Zuur et al. (2009). All models for outliers and residuals for homogeneity were checked; no problems were found.

Prior to quality analysis, functions ‘rawplot.electrophoresis’, ‘integrate.custom’ from the R package bioanalyzeR were used to extract fragment distribution data from the TapeStation metadata XML and electropherograms in CSV outputs. After this, TapeStation results yielded concentrations of fragments in three size classes (1–10 kbp: Small, 10–20 kbp: Medium and 20–48.5.5 kbp: Large). We present means and 95% confidence intervals for each of these. For statistical analysis, we chose to focus on the 20–48 kbp size class given its importance for advanced sequencing applications. For each sample we calculated the proportion of gDNA in this class and analysed these data using beta regression and a logit link.

For DNA composition, data represented counts of bacterial DNA copies which were analysed with simple linear regression. We report means and 95% confidence intervals and contrast with gDNA concentration.

Finally, we evaluated efficacy of the Qiagen MagAttract DNA kit relative to the Qiagen DNeasy Blood & Tissue with just one tissue treatment (Whole-Sliced). We followed the same analyses as described above for the larger datasets but with a simple two-level factor of extraction kit type to evaluate evidence for differences. The modest size of our dataset (*N* = 6 MagAttract HMW Kit and *N* = 3 DNeasy Blood & Tissue Kit) meant that detecting differences was unlikely and we were cautious in our inference.

## Results

### Microscopy

Under UV light, the tick sections exhibited pronounced autofluorescence, complicating the visualisation of stained DNA using DiamondTM Nucleic Acid Dye (Promega) (Fig. 2a). When employing H&E stain under visible light however, a sparse presence of nuclei was observed in the leg tissue, in which a large proportion is made up by exoskeleton (Fig. 2b). In contrast, the abdomen, especially the gut region, exhibited a notably higher concentration of cell nuclei (Fig. 2c).

### DNA quantification

#### Tissue type and disruption

**Fig. 3:**
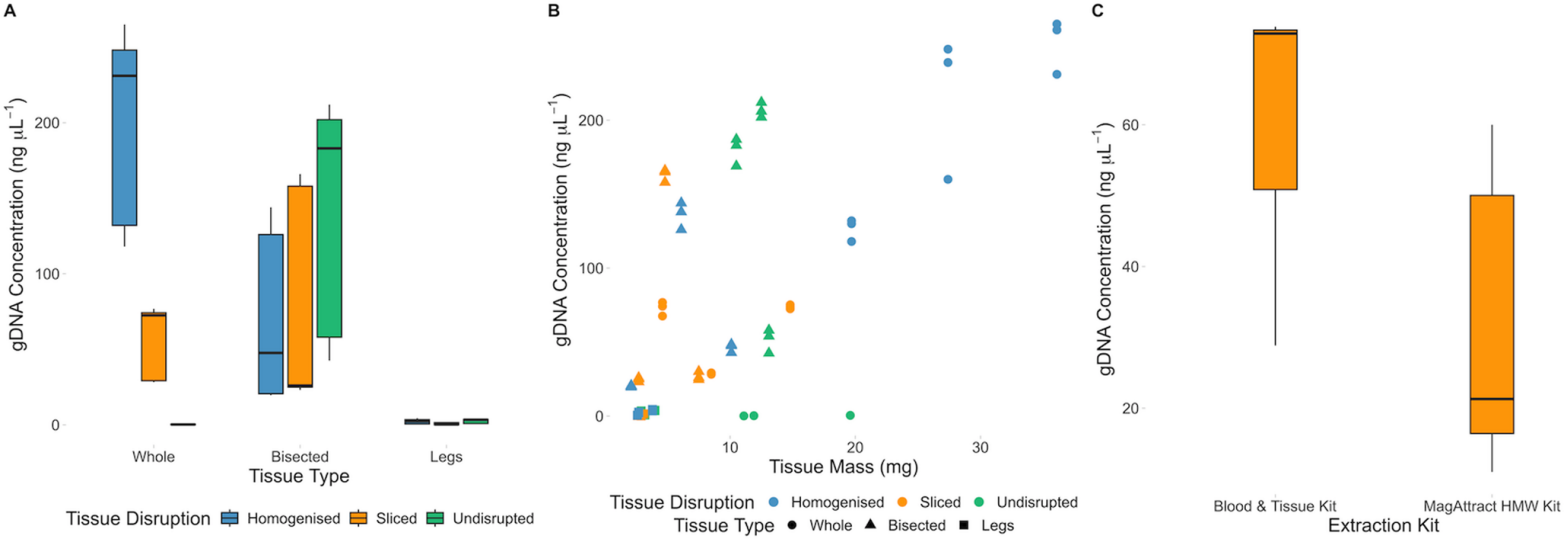
**A**: DNA concentration yield of each tissue treatment. **B**: Comparison of DNA concentration yield and the mass of the input tissue. **C**: DNA concentration yield of Whole-Sliced samples using Qiagen DNeasy Blood & Tissue Kit and Qiagen MagAttract HMW Kit

The highest DNA concentrations were associated with Whole-Homogenised samples (198 ng µL^− 1^, SE = 20.6). Whole-Sliced samples yielded less (58.5 ng µL^− 1^, SE = 7.47 Fig. 3A) but the effect is not significant (Table 3). Bisected-Undisrupted samples yielded similar quantities compared to Whole-Homogenised (146 ng µL^− 1^, SE = 24.0, Fig. 3A) despite the mean weight of Bisected-Undisrupted tissue being less (12.0 vs. 27.7 mg respectively, Fig. 3B). Additionally, Bisected-Sliced samples yielded less than Bisected-Undisrupted (71.5 ng µL^− 1^, SE = 22.9). Whole-Undisrupted and Legs (all tissue disruption methods) treatments yielded substantially less DNA overall (Whole-Undisrupted: mean = 0.250 ng µL⁻¹, SE = 0.0539; Legs: mean = 1.87 ng µL⁻¹, SE = 0.290). Both Whole-Undisrupted and Legs-Undisrupted samples produced significantly lower yields (Whole-Undisrupted: estimate = − 149, SE = 55.4, t = − 2.68, *p* = 0.0159; Legs-Undisrupted: estimate = + 148, SE = 68.6, t = 2.15, *p* = 0.046; see Table 3). In some treatments, tissue disruption and tissue type had clear interactions (Table 3) reflecting the contingent nature of the amount of tissue in each tissue type and its preparation. Legs and Whole-Undisrupted specimens clearly yielded an inadequate quantity of DNA for current and advanced sequencing technologies using presently accessible extraction kits, while Bisected-Undisrupted stood out in producing the highest quantity of DNA relative to mass of input tissue (Fig. 3B).

**Table 3:** The values in this table are estimated DNA concentrations (ng/µL) produced by a linear mixed-effects model that accounts for tissue type, disruption method, and extraction kit. The numbers are model predictions with their associated standard errors (± SE), not raw measurements. They represent the expected change in DNA yield under each condition according to the model

Factor	Estimate	SE	t Value	*p* Value
*Intercept - Whole-Homogenised*	96.9	84.6	1.14	0.268
*Bisected*	−51.7	73.8	−0.70	0.493
*Legs*	−106	81.4	−1.30	0.211
*Sliced*	−72.4	66.1	−1.09	0.289
*Undisrupted*	−149	55.4	−2.68	0.0159
*Tissue Weight (mg)*	3.65	2.88	1.27	0.222
*Bisected : Sliced*	80.4	74.8	1.07	0.298
*Legs : Sliced*	71.3	76.7	0.930	0.365
*Bisected : Undisrupted*	205	79.1	2.60	0.0188
*Legs : Undisrupted*	148	68.6	2.15	0.0460
**Extraction Kits**				
*Intercept - Blood & Tissue*	35.7	25.8	1.39	0.215
*MagAttract HMW*	−39.0	20.0	−1.96	0.0982
*Tissue Weight (mg)*	2.46	2.37	1.04	0.340

#### Extraction kits

Whole-Sliced samples were used to compare the yields generated by the Qiagen DNeasy Blood & Tissue kits and MagAttract HMW DNA Extraction Kits (Qiagen, Germany). The Qiagen DNeasy Blood & Tissue kit produced higher mean yields than the MagAttract kit (58.5, SE = 7.47 vs. 38.8 ng µL^− 1^, SE = 9.05; Fig. 3C) but with the difference not being significant in using the MagAttract kit (est. = −39.0, SE = 20.0, t = −1.96, *p* = 0.0982; Table 3).

### DNA quality

#### Tissue type and disruption

**Fig. 4:**
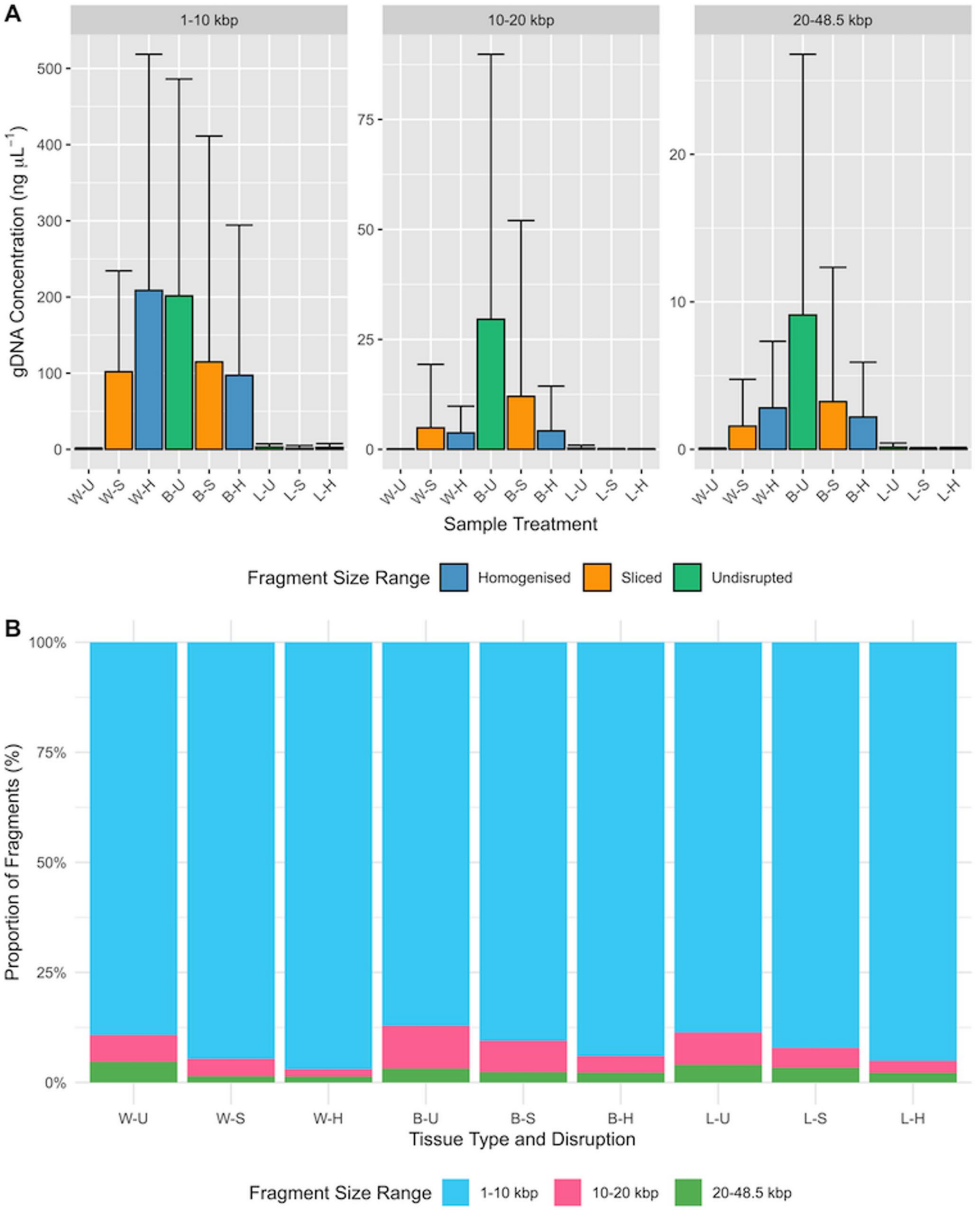
**A**: Mean concentration of gDNA present of each fragment range (1–10 kbp, 10–20 kbp and 20–48.5.5 kbp) found in each sample treatment. **B**: Mean proportion of each fragment range between different sample treatments. All samples processed using Qiagen DNeasy Blood & Tissue Kit and in triplicate. Key: W = Whole, B = Bisected, L = Legs, U = Undisrupted, S = Sliced, H = Homogenised

The greatest concentration of large fragments was generated by the Bisected-Undisrupted samples (9.11 ng µL^− 1^, SE = 4.11; Fig. 4A), with the interaction effect of using treatments Bisected and Undisrupted increasing from the reference point of Whole-Homogenised (est. = −4.26, SE = 0.199, z = −21.4, p = < 0.001; Table 4). Whole-Homogenised, however, the standard practice, yielded a lower concentration of large fragments (2.81 ng µL^− 1^, SE = 1.05). Variation in mean proportion of large DNA fragments between tissue treatments was marginal, ranging between 0.0131 and 0.0469 (Fig. 4B).

**Table 4:** ß distribution effects model of proportion of large fragment size (20–48.5.5 kbp) variation between different tissue types and extraction kits

Factor	Estimate	SE	z Value	*p* Value
*Intercept - Whole-Homogenised*	−4.26	0.199	−21.4	< 0.001
*Bisected*	0.509	0.254	2.01	0.0446
*Legs*	0.478	0.255	1.87	0.0611
*Sliced*	0.0220	0.280	0.0785	0.937
*Undisrupted*	1.26	0.228	5.53	< 0.001
*Bisected : Sliced*	0.0328	0.356	0.0922	0.927
*Legs : Sliced*	0.389	0.348	1.12	0.264
*Bisected : Undisrupted*	−1.04	0.312	−3.34	< 0.001
*Legs : Undisrupted*	−0.658	0.304	−2.17	0.0302
Extraction Kits				
*Intercept - Blood & Tissue*	−3.97	0.412	−10.1	< 0.001
*MagAttract HMW*	2.28	0.424	5.38	< 0.001

As with the qubit measurements, Leg (all tissue disruptions) and Whole-Undisrupted samples had the least concentration of large fragments (0.0461–0.152 ng µL^− 1^; Fig. 4A). Additionally, there was a marginal model effect on the proportion of large fragments (Legs: est. = 0.478, SE = 0.255, z = 1.87, *p* = 0.0611; Undisrupted: est. = 1.26, SE = 0.228, z = 5.53, p = < 0.001; Table 4).

The greatest concentration of small fragments was produced by Whole-Homogenised treatments (209 ng µL^− 1^, SE = 72.0; Fig. 4A). followed closely by Bisected-Undisrupted (202 ng µL^− 1^, SE = 66.1). Legs (all tissue disruptions) and Whole-Undisrupted produced the least concentration of small fragments, ranging between 1.16 and 3.05 ng µL^− 1^.

Beta analysis revealed that Whole-Homogenised samples had a statistically significant negative effect on the proportion of large fragments (est. = −4.26, SE = 0.199, z = −21.4, p = < 0.001; Table 4). As did the interaction effects of Bisected-Undisrupted and Legs-Undisrupted, but less so (Table 4). Bisected and Undisrupted as isolated factors had a marginal positive effect of large fragments with statistical significance (Bisected: est. = 0.509, SE = 0.254, z = 2.01, *p* = 0.0446; Undisrupted: est. = 1.26, SE = 0.228, z = 5.53, p = < 0.001 Table 4).

#### Extraction kits

**Fig. 5:**
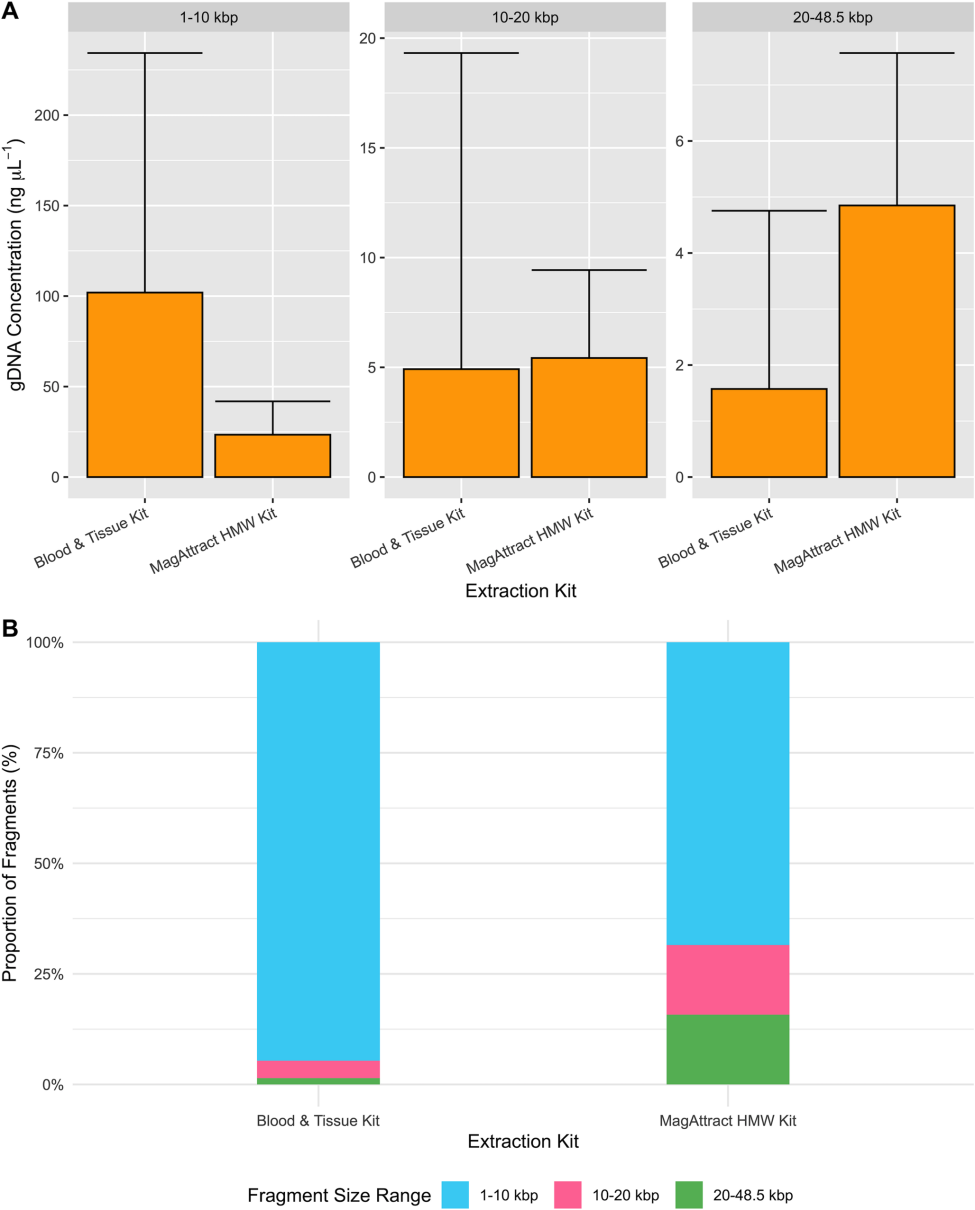
**A**: Mean concentration of gDNA present of each fragment range (1–10 kbp, 10–20 kbp and 20–48.5.5 kbp) found between Whole-Sliced specimens with DNA purified from Qiagen DNeasy Blood & Tissue Kit or Qiagen MagAttract HMW Kit. **B**: Mean proportion of each fragment range between Whole-Sliced samples extracted using Qiagen Blood & Tissue Kit (*n* = 3) or Qiagen MagAttract HMW Kit (*n* = 6)

Samples extracted using the MagAttract HMW kit had a greater concentration of large fragments (4.85, SE = 1.06 vs. 1.57 ng µL^− 1^, SE = 0.739; Fig. 5A). This is also true when considering the proportion of large fragments observed within MagAttract HMW kit samples, a proportion of 0.157, vs. 0.0142 for the DNeasy Blood & Tissue kit (Fig. 5B). Beta regression analysis revealed a statistically significant increase in the proportion of large fragments (est. = 2.28, SE = 0.424, z = 5.38, p = < 0.001; Table 4) and a reduction in the proportion of short fragments (est. = −2.06, SE = 0.362, z = −5.67, p = < 0.001) by using the MagAttract HMW Kit.

### DNA composition

#### Tissue type and disruption

**Fig. 6:**
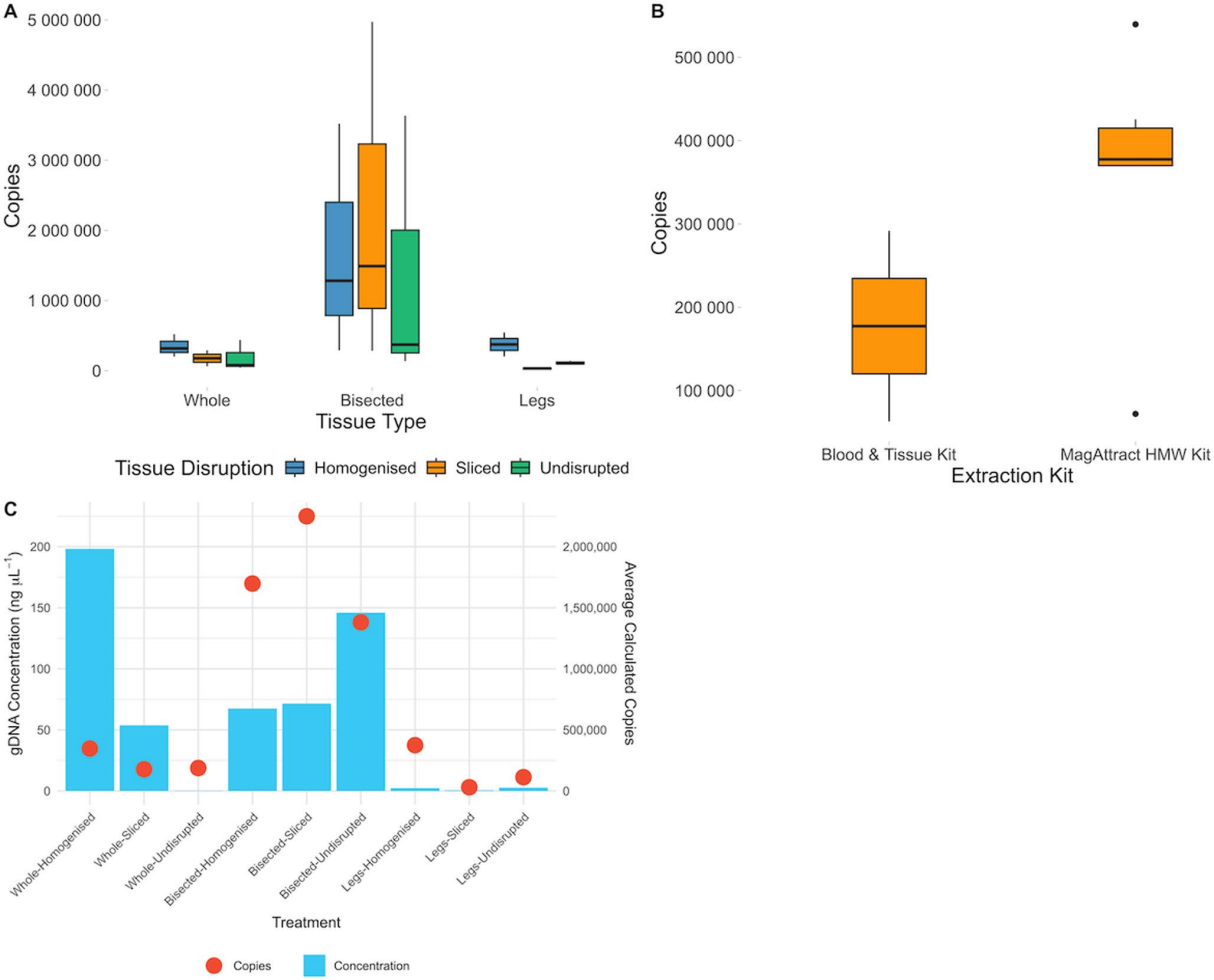
**A**: Number of bacterial copies obtained from different tissue type and preparation treatments. **B**: Number of bacterial copies obtained from different extraction kit methods. **C**: Comparison of gDNA concentration (ngµL-1) (bar chart) overlayed with average number of bacterial copies (dots) per sample treatment

Legs-Sliced had the lowest bacterial copy number (mean 30,223 copies) in contrast to Bisected-Sliced which had the highest bacterial copy number (avg. 2,248,966 copies). Legs produced the lowest mean bacterial copy number followed by Whole and then Bisected, with exception of Legs-Homogenised which was similar to Bisected treatments (Fig. 6A).

Regression revealed no significant effect of treatments on copy number (Table 5). The greatest positive effect was from Bisected (est. = 1,351,139, SE = 1,024,877, t = 1.32, *p* = 0.206; Table 5). The greatest negative effect was from the interaction effect of Legs : Sliced (est. = −174,789, SE = 1,620,472, t = −0.108, *p* = 0.915; Table 5).

**Table 5:** Generalised linear model analysis of qPCR copy number using different tissue Types, disruption methods and extraction kits

Factor	Estimate	SE	t Value	*p* Value
*Intercept – Whole-Homogenised*	346,980	724,697	0.489	0.639
*Bisected*	1,351,139	1,024,877	1.32	0.206
*Legs*	27,586	1,145,847	0.0241	0.981
*Sliced*	−169,554	1,145,847	−0.148	0.884
*Undisrupted*	−159,370	1,024,877	−0.156	0.878
*Bisected : Sliced*	720,401	1,537,315	0.469	0.646
*Legs : Sliced*	−174,789	1,620,472	−0.108	0.915
*Bisected : Undisrupted*	−157,593	1,449,395	−0.109	0.915
*Legs : Undisrupted*	−102,636	1,537,315	−0.0668	0.948
**Extraction Kits**				
*Intercept - Blood & Tissue Kit*	177,426	110,484	1.61	0.159
*MagAttract HMW Kit*	182,894	127,576	1.43	0.202

#### Extraction kits

Comparing the bacterial load between extraction kits, the Qiagen DNeasy Blood & Tissue kit had the lowest bacterial copy number (mean of 177,426 copies) compared to Qiagen MagAttract which had a higher bacterial copy number (mean of 320,351 copies).

As with the tissue type and disruption, after performing a generalised liner model, it was found that changing extraction kit did not cause a statistically significant effect. Additionally, the estimated change by using each factor was marginal (Table 5).

## Discussion

Obtaining high yields of high molecular weight DNA is critical for advanced sequencing methods. When tested independently, Bisected ticks with no tissue disruption and the Qiagen MagAttract HMW kit each provided improved DNA quality and quantity over other treatments tested, indicating that these choices would be suitable for advanced sequencing applications.

Unoptimised DNA extractions may result in wasted time, finances, and material resources due to the need to troubleshoot methodologies. Here, we provide guidelines on the best ways to obtain optimal input DNA which is essential for advanced sequencing technologies (Oosting et al. 2020). Getting the correct DNA quality and quantity can be troublesome, particularly for low biomass multiple DNA source vector arthropods like ticks, and these difficulties are rarely reported in the literature.

Some treatments revealed clear trade-offs between DNA yield and fragmentation (Whole, Legs, Undisrupted), but others possessed few trade-offs (Bisected), generating appropriate extractions for a wide range of sequencing technologies. Further, the use of Legs and Whole-Undisrupted treatments may be required for some museum specimens, but these sample types had very poor potential across the board for advanced sequencing platforms. Microscopy confirmed these observations with very few observable nuclei housed within the exoskeleton, which comprises most of the leg tissue in ticks. This means that studies where only limited tissue can be recovered may not be suitable for more advanced sequencing platforms but may still be suitable with short PCR amplicons (i.e. COI).

### Optimising quantity

For maximum DNA yield, using an entire specimen coupled with bead homogenisation is preferable, in keeping with a previous study comparing non-destructive and destructive DNA extractions in spiders (Paquin and Vink 2009). Bead beating homogenises the specimen, increasing the lysis buffer’s surface contact, allowing it to more easily infiltrate cells, improving DNA extraction efficiency (Marquina et al. 2022). Conversely, a whole specimen left undisrupted yielded a negligible amount of DNA (mean of 0.250 ng µL^− 1^). This is likely because the lysis buffer cannot penetrate the chitinous exoskeleton, which can be alleviated by puncturing the exoskeleton though with potential trade-off of damaging the specimen (Phillips and Simon 1995).

A balanced integration of both methods involves simply bisecting the specimen, a technique that produces practical quantities of DNA for most applications, giving access to tissues and avoiding the need for tissue disruption via slicing. Results demonstrate that slicing a Bisected specimen yields 71.5 ng µL^− 1^, compared to 146 ng µL^− 1^ for an Undisrupted-Bisected specimen. As well as the mean yield being lower, the extraction workflow merits consideration. Slicing the specimen into 1–2 mm fragments adds an additional 1–1.5 min of preparation time (slicing with scalpel blade and sterilisation) and additional cost per sample. Additionally, tissue and cellular fluids lost on the scalpel blade and cutting surface during slicing may not be transferred to the lysis buffer for digestion, potentially contributing to reduced DNA recovery. This increase in time becomes substantial when processing many samples. Given the significant increase in effect of DNA yield (+ 205.30 ng µL^− 1^; Table 3) when using undisrupted bisected specimens compared to undisrupted sliced specimens, slicing specimens is not justified to maximise DNA yield.

Extracting DNA solely from tick legs yielded insufficient DNA concentrations (mean of 0.638–2.65 ng µL^− 1^), rendering them inadequate for most modern sequencing applications. This includes long-read sequencing protocols like those reported by Kingan et al. (2019), where attempting to use DNA inputs as low as 100 ng for PacBio Single Molecule Real-Time sequencing was challenging, with the addition of being an unpractical workflow for efficient sample processing (Kingan et al. 2019). However, tick legs may be feasible for PCR focused applications which only requires around 1–100 ng of purified gDNA for successful amplification (Abellan-Schneyder et al. 2021). Yet with the increase in processing time to remove the tick legs and drastic reduction in DNA yield, using tick legs is not recommended for optimising DNA quality.

This study revealed that Qiagen MagAttract HMW kit produced less DNA (mean of 38.8 ng µL^− 1^) than the Qiagen DNeasy Blood & Tissue kit (mean of 58.5 ng µL^− 1^) though the differences were not significant. However, both methods produced sufficient concentrations of DNA for PCR and advanced sequencing applications. If maximising quantity needs to be prioritised, given its decrease in cost and more user-friendly workflow, the Qiagen DNeasy Blood & Tissue kit is recommended over Qiagen MagAttract HMW kit for use in this context.

### Optimising quality

For applications requiring HMW DNA with minimal shorter fragments, a kit designed for HMW purification, such as the Qiagen MagAttract HMW Kit, is recommended as it produced the fewest 1–10 kbp fragments and the most 20–48.5.5 kbp fragments (Figs. 4 and 5). Combining this kit with minimally destructive tissue preparation techniques that also provide an appropriate quantity of DNA will maximise the yield of large fragments (20–48.5.5 kbp). This includes Whole and Bisected tissue types and Undisrupted or Sliced methods. Though the differences between these tissue preparations were minimal resulting in a difference in the proportion of large fragments being no more than 0.0337 between the highest (Whole-Undisrupted) and lowest (Whole-Homogenised) of these tissue preparations. Despite a higher yield of overall DNA, aggressive homogenisation results in shorter DNA fragments, i.e. gDNA is mechanically sheared. This is evident as whole-homogenised samples produced the largest proportion of small fragments (0.97 of 1–10 kbp), a method widely reported for extracting DNA from ticks (Kelly et al. 2014; Guerrero et al. 2019). To maximise DNA fragment quality, it is therefore recommended to avoid bead-beating homogenisation for small arthropods.

Differences in the proportion of large fragments are more obvious when comparing extraction kits (Fig. 5B), with the Qiagen MagAttract Kit producing a higher proportion of large fragments (0.158) and a lower proportion of short fragments (0.685). This suggests that the type of extraction kit or system will be more impactful than changes in tissue preparation, excluding Legs and Whole-Undisrupted. DNA purifications to optimise HMW recovery (in which the kit choice was most impactful) are particularly suitable for long-read applications, including PacBio Sequel II, Oxford Nanopore, and ddRADseq library development (Jain et al. 2016; *Technical Note: Preparing DNA for PacBio HiFi Sequencing — Extraction and Quality Control* 2022). Although minor differences were observed in the proportion of large fragments between treatments, the majority of fragments remained small across all treatments. This indicates that further purification or enrichment may be required to obtain longer fragments for long-read applications such as PacBio or Oxford Nanopore sequencing; however, such steps are commonly incorporated into standard library preparation workflows.

### Optimising composition

Legs-Sliced samples produced the lowest bacterial copy number (30,222 copies), which is expected given the small quantity of total DNA produced. Due to the difficulty in obtaining a usable quantity of DNA from tick legs alone, it may be more beneficial to consider alternative methods, such as removing the gut, despite an increased processing time. Alternatively, the presence of the microbiome in a sample may be advantageous, using Whole samples with an intact microbiome allows extracted DNA to be utilised in further applications, such as microbiome analysis (Egan et al. 2021). However, if reduction in microbial load is required, Legs produced similar bacterial counts to Whole samples. Despite having a higher quantity of DNA extracted, Whole samples resulted in only a slightly higher bacterial copy number (Fig. 6C), further showing the ineffectiveness of using legs for DNA extractions.

Bisected tissue type resulted in the greatest increase in bacterial copy number (Fig. 6A). This likely occurs because the full contents of the specimen gut are exposed to the lysis buffer for the longest period of time. However, the negligible difference in bacterial copy number between Whole specimens and Legs, despite differences in DNA yield and the presence/absence of gut tissue, suggests that regulating microbial load will be challenging. Therefore, it may be more effective to extract a larger quantity of DNA using Whole or Bisected tissue types and subsequently remove unwanted bacterial sequences bioinformatically during the analysis pipeline (Wang et al. 2022), by employing techniques such as adaptive sequencing on Oxford Nanopore (Ulrich et al. 2022) or performing targeted amplicon sequencing.

### Tick microscopy

The initial aim of this study was to determine the spatial distribution of DNA within a tick specimen, with a particular focus on DNA presence within the exoskeleton. However, the autofluorescence exhibited by tick sections under UV light posed significant challenges in visualising DNA using UV fluorescent dyes such as SYBR Green. This issue is consistent with previous findings in tick specimens (Shade et al. 2018). Consequently, studies that rely on UV fluorescence for visualising tick structures should be cautious of potential false positives due to the inherent autofluorescence of tick tissues. Diamond Dye is a universal DNA binding dye, so targeted probe-based assays (i.e. fluorescent in-situ hybridisation), or dyes with emission in a different wavelength, may be better suited to this task.

## Conclusions

Sequencing tools are rapidly evolving to investigate increasingly complex biological questions. This makes optimisation of gDNA purifications imperative, as advanced sequencing technologies require more stringent input requirements in terms of DNA quantity and quality (Jaudou et al. 2022). However, most published literature only tests extraction success via PCR. PCR requires only low quantities of DNA that can be highly fragmented, meaning it cannot adequately assess whether DNA quality is sufficient for advanced sequencing applications (Halos et al. 2004; Ammazzalorso et al. 2015).

This study demonstrates that non-aggressive tissue disruption combined with a HMW-tailored DNA extraction kit is optimal for obtaining HMW DNA. For maximum DNA yield, homogenising an entire specimen is recommended. However, bisecting a specimen can also provide sufficient DNA for most applications while preserving excess tissue for future use. Extracting DNA solely from tick legs is inadequate for most purposes due to the overall low biomass and DNA content. In terms of DNA bacterial load, bisected specimens produced the highest copy numbers, with whole specimens and legs producing the least. We acknowledge that the small sample size may have contributed to higher standard errors in some analyses. Nonetheless, by using mixed effects modelling, we were able to account for individual variation and improve the reliability of our estimates.

By systematically testing tissue type, disruption technique, and extraction kit, and measuring DNA purification success through quality, quantity, and bacterial load, this study ensures efficient use of resources and successful extractions for target applications, particularly in complex multiple DNA source samples such as ticks. Additionally, this study provides a framework for future research to report extraction optimisations accounting for gDNA quantity, quality, and composition.

The methods and results presented in this paper are broadly applicable to a wider range of arthropod taxa, particularly those of similar size to *A. triguttatum.* Researchers can adapt the techniques based on factors such as exoskeleton thickness (e.g., mosquitoes vs. beetles). Future studies optimising DNA extraction should consider the quantity, quality, and composition of the extracted DNA, as highlighted in this study. Future studies optimising arthropod DNA purifications would benefit from testing how factors such as storage temperature, specimen age, and lysis time affect the quality, quantity, and composition of output DNA. Continued optimisation of gDNA extraction methods will yield high-quality DNA, facilitating the generation of reliable data for current and emerging advanced sequencing applications across a broad range of arthropod taxa.

## Data Availability

The data that support the findings of this study are available from the corresponding author upon reasonable request.
